# Mucous Extravasation Cyst Associated With the Lower Lip and Its Management: A Case Report

**DOI:** 10.7759/cureus.46557

**Published:** 2023-10-05

**Authors:** Unnati Shirbhate, Pavan Bajaj, Manoj Patil, Sneha Dare

**Affiliations:** 1 Department of Periodontics, Sharad Pawar Dental College, Datta Meghe Institute of Higher Education and Research, Wardha, IND; 2 Department of Research and Development, Datta Meghe Institute of Higher Education and Research, Wardha, IND

**Keywords:** salivary duct, mucous extravasation cyst, lower lip, scalpel excision, salivary gland, mucocele

## Abstract

A 15-year-old female patient was reported with swelling on the right side of the lower lip for 15 days. A provisional diagnosis of mucocele was obtained based on the patient's history and clinical examination. Under all aseptic conditions and administration of local anesthetic, surgical mucocele removal was done using a scalpel. An excised soft tissue specimen was given for histopathological examination, confirming the final diagnosis of mucous extravasation phenomena or mucocele. Recall examination after seven days reveals satisfactory lesion healing and no discomfort. This case report demonstrates that surgical excision is a simple, efficient, and affordable method for treating mucoceles and giving aesthetic and functional clearance.

## Introduction

The most frequent lesion of the oral mucosa is called a mucocele, and it is brought on by an accumulation of mucus secretion caused by trauma, lip-biting habits, or minor alterations in the salivary glands [1]. According to histological characteristics, they primarily fall into retention mucoceles (RMs) and extravasation mucoceles (EMs). Retention-type mucoceles are incredibly uncommon, while extravasation mucoceles are frequently observed. Extravasation mucocele, which affects small salivary glands, is brought on by fluid oozing from the compromised salivary gland ducts and acini into the nearby soft tissues. Three stages of evolution are present in these extravasation mucoceles [2]. The mucus diffusely leaks into the connective tissues from the excretory duct in the initial stage. A foreign body reaction causes granuloma to form during the second resorption phase. The creation of a pseudo-capsule surrounding the mucosa occurs in the final step. Blockages of the major salivary gland ducts are common and cause retention-type mucoceles [3]. The lip is usually affected by mucoceles, but they may develop anywhere in the oral mucosa, including the cheeks and floor of the mouth.

The basic foundation for a diagnosis is the clinical examination of the patient. Extravasation mucocele most usually occurs on the lower lip. Mucoceles most frequently affect young individuals, yet they can affect people of any age [4]. They could be translucent cystic swellings with a bluish appearance and a soft consistency. Mucocele typically manifests as an asymptomatic vesicle or bullae that is pink or bluish. It can range in size from 1 mm to several centimeters, and it can affect both genders and people of all ages, with the peak occurrence occurring between 10 and 20 years of age [5]. The standard course of treatment involves surgically removing the epithelial and mucosal tissue beneath the muscular layer of the mucocele. The mucocele could be easily removed, allowing the content to drain out, but the lesion would relapse as soon as the wound healed. Treatment options include surgical excision and removal of the affected accessory salivary gland. Marsupialization will only cause recurrence [6]. Patients are sensitive to the affected mucocele area; they can be uncomfortable even though most are painless. Mucoceles can obstruct speech or chewing. Shallow mucoceles can rupture and leak straw-colored fluid. Deeper ones have a longer life span and a higher propensity to cause patient discomfort [5,6].

## Case presentation

**Figure 1:**
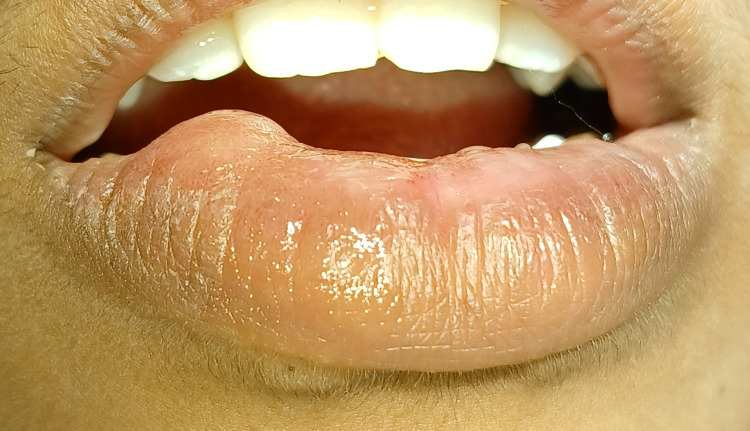
Preoperative view of the mucocele associated with the lower lip

A 15-year-old female patient was reported to the department of periodontics with the chief complaint of 10- to 12-mm circular swelling on the right side of the lower lip for 15 days, which was initially small and progressed later. Considering the history of trauma, lip-biting, and clinical signs on examination, the lesion was soft, fluctuant, palpable, and classified as a mucocele. Routine hematological tests revealed results that were within normal range. Mucocele, lipoma, traumatic fibroma, and fibrous hyperplasia were among the differential diagnoses. A provisional diagnosis of mucocele was obtained based on the patient's history and clinical examination. Figure 1 shows a preoperative view of the right-side lower lip mucocele, with the lesion measuring up to 1 cm wide and 1 cm long. The patient could not close her lips and felt discomfort and pain at the site.

**Figure 2:**
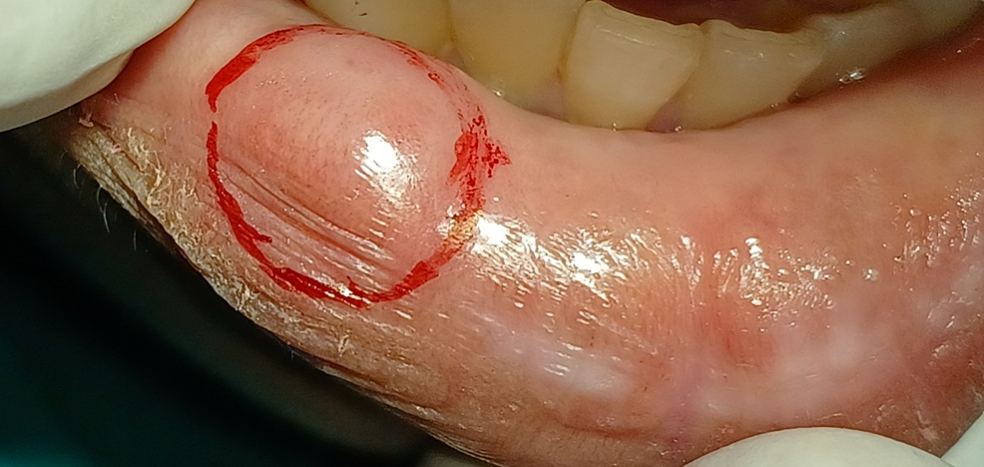
Markings done prior to surgical removal

The patient was informed about the surgical removal of the mucocele, and written consent was obtained from the patient and her parents. Under all asepsis and administration of local anesthetic, markings were done around the border of the mucocele lesion, which can be seen in Figure 2.

**Figure 3:**
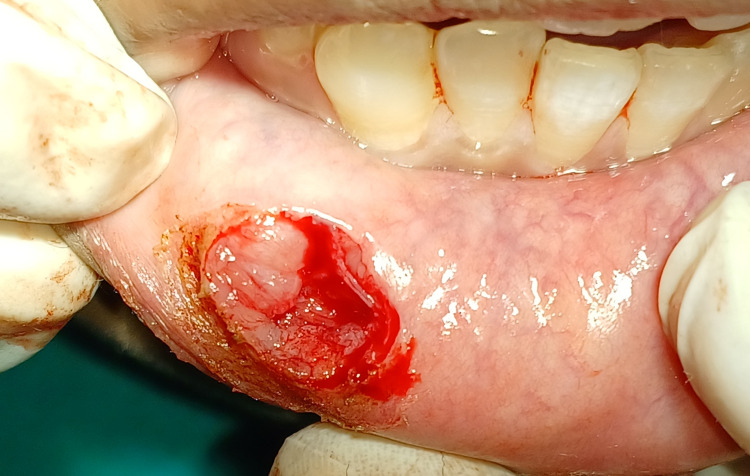
Operated site

The surgical removal of the mucocele was done using a scalpel by resecting it from the base to reduce the chances of reoccurrence, as shown in Figure 3.

**Figure 4:**
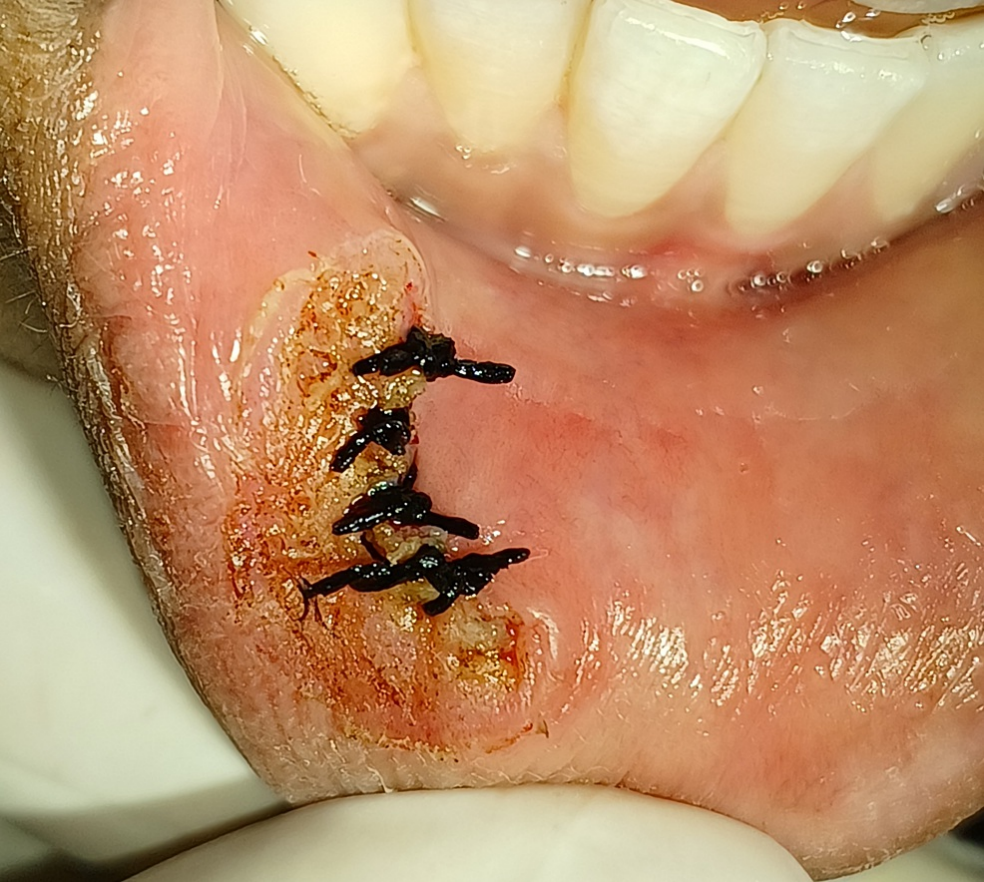
Sutures placed

Sutures were placed after achieving hemostasis, as shown in Figure 4.

**Figure 5:**
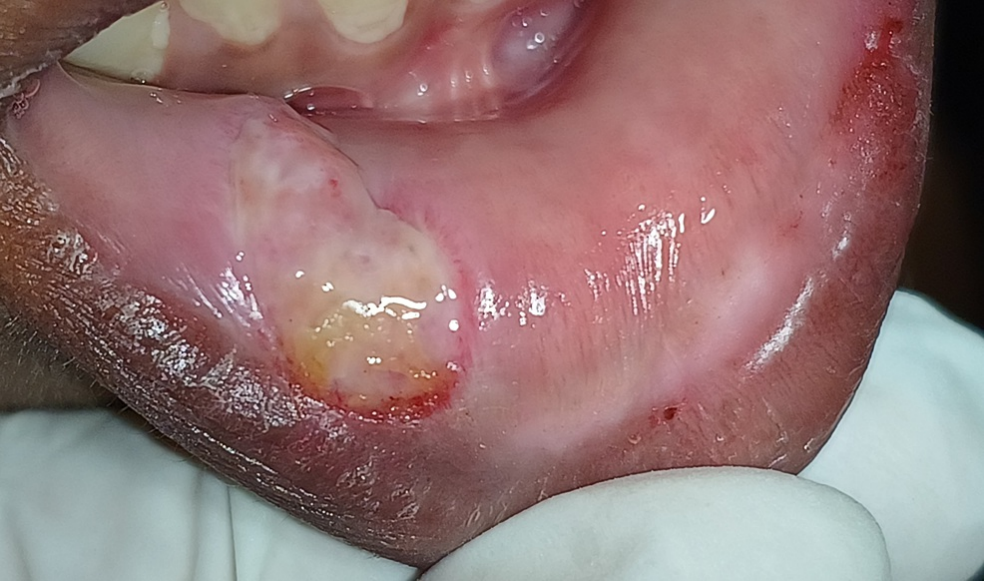
Sutures removed after seven days revealing satisfactory healing of the operated site

Postoperative instructions and medications were given. The patient was recalled for evaluation and suture removal after seven days, which revealed satisfactory wound healing, as shown in Figure 5.

**Figure 6:**
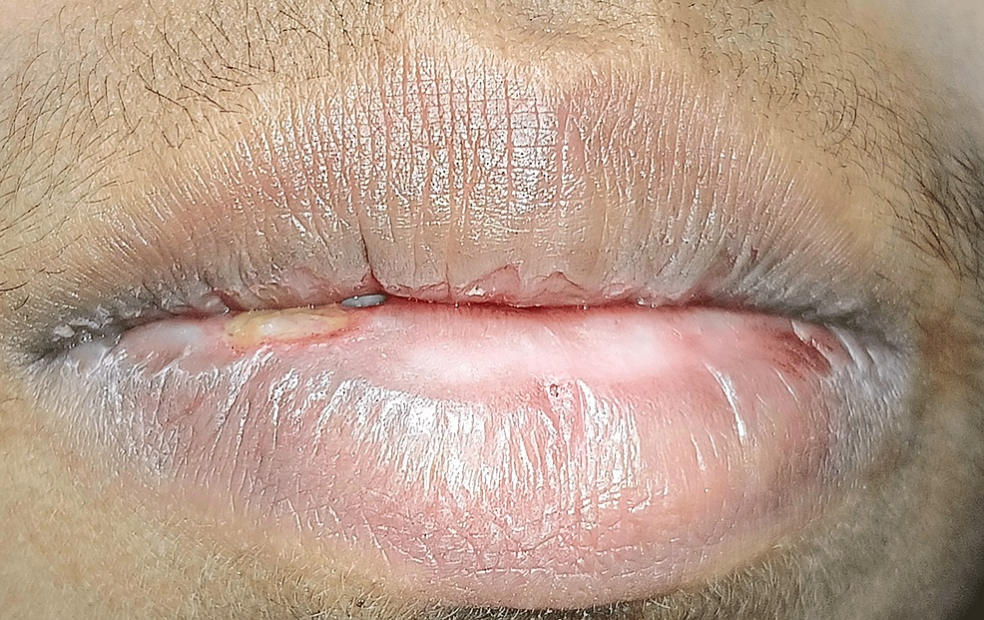
Postoperative view of the patient showing satisfactory healing, as well as no functional problem

The patient could close her lips, as appreciated in Figure 6, postoperatively after seven days. No discomfort or pain was reported.

**Figure 7:**
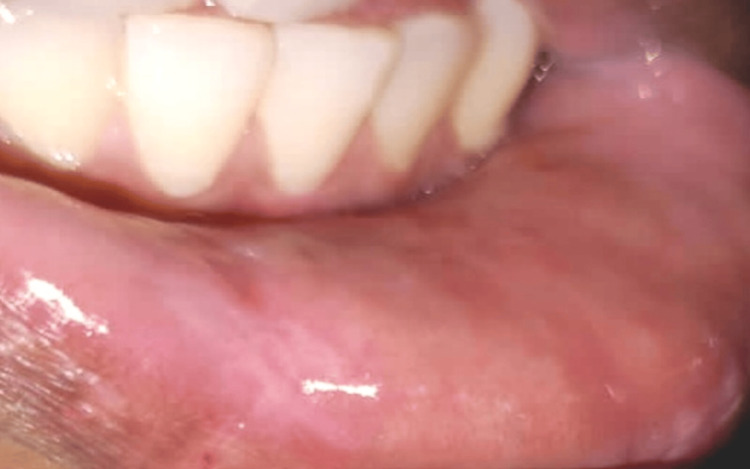
Postoperative follow-up after one month revealing satisfactory healing and no recurrence and scar formation

Postoperative recall examination of the patient after one month revealed good wound healing and no recurrence, despair, or scar formation at the site as shown in Figure 7.

**Figure 8:**
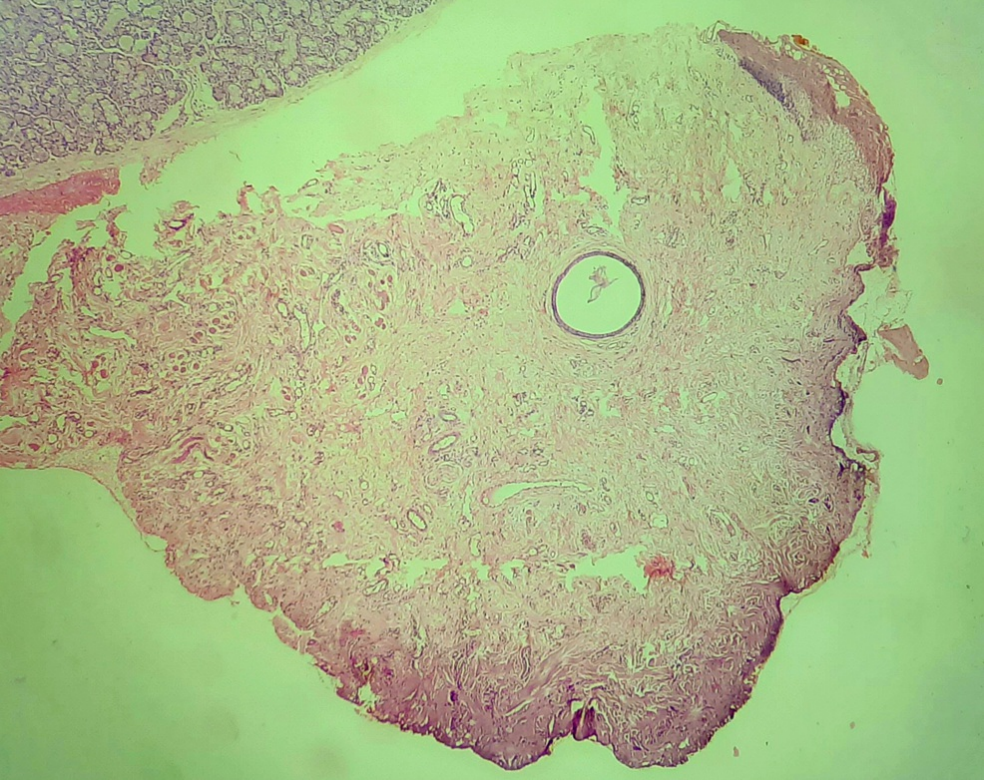
Histopathological examination of soft tissue specimen showing mucous acini, granulation tissue, and mucous pooled area, which confirms mucous extravasation phenomenon

An excised soft tissue specimen was given for histopathological examination, confirming the final diagnosis of mucous extravasation phenomena or mucocele. Figure 8 shows a histopathological diagram of the mucocele where the mucous acini, mucous pooled area, and granulation tissue are present. The patient is reviewed for the next three months, showing no recurrence and complete recovery of the mucocele.

## Discussion

In the general population, the prevalence of mucocele is between 0.4% and 0.9% [3]. Mucocele has no gender predilection. The pathognomonic sign of mucocele is its appearance. The diagnosis of superficial mucocele is made based on the location of the lesion, history of trauma, quick emergence, variability in size, bluish color, consistency, history, and clinical signs. Lips contain fatty tissue, connective tissue, blood vessels, nerves, and salivary glands; hence, swelling on the lips can result from disease of any of these tissues. Lip swelling is a symptom of mucocele, fibroma, lipoma, mucus retention cyst, sialolith, phlebolith, and salivary gland tumor. However, these can be separated from mucoceles based on their clinical characteristics, color, consistency, etiology, and place of occurrence [3,5]. The most typical treatment approach for mucocele is traditional surgical removal. The therapeutic method that is most frequently employed is elliptical incision. This lessens the amount of mucosal tissue lost, lowers the likelihood that extensive fibrous scars will form, and helps prevent cystic contents from spilling, which could lead to recurrence [1,6].

Mucoceles are benign lesions. They can, however, organize to develop a persistent bump on the oral surface if not treated. Wherever there are salivary glands, mucoceles develop [7]. Extravasation mucoceles (EMs) and retention mucoceles (RMs) are two different types of mucoceles that can be created. While RM is highly uncommon, EM is typical in children. A retention mucocele develops when a sialolith or ductal scar blocks the salivary duct, and the mucin is subsequently encircled by ductal epithelium. While RM is connected to the ductus' traumatic injury, EM results from saliva extravasating into the nearby connective tissue regions [8]. The most common methods to remove a recurrent or chronic mucocele are surgical excision, cryosurgery, diode laser therapy, and electrocautery. Among the many techniques, traditional surgical excision was deemed to be a simple, efficient, and affordable method for treating mucocele in kids. Surgical removal was considered a treatment option for the present case, as swelling was associated with functional problems [9].

## Conclusions

Recall examination after seven days revealed satisfactory healing of the mucocele lesion, less scarring, and no discomfort and pain. The patient is reviewed for the next three months, showing no recurrence, no scar formation, and complete recovery of the mucocele. Patient discomfort and aesthetic appearance at the site of the patient's lesion are improved in this sensible case presentation. What makes this case unique is achieving remarkable final aesthetic results after surgery and no recurrence sign with follow-up examination. This case report demonstrates that surgical excision is a simple, efficient, and affordable method for treating mucocele and the functional problems associated with mucocele, which gives patients confidence and remarkable aesthetic outcomes.
